# Polychlorinated Biphenyls Disrupt Blood–Brain Barrier Integrity and Promote Brain Metastasis Formation

**DOI:** 10.1289/ehp.0901334

**Published:** 2009-10-28

**Authors:** Melissa Seelbach, Lei Chen, Anita Powell, Yean Jung Choi, Bei Zhang, Bernhard Hennig, Michal Toborek

**Affiliations:** 1 Molecular Neuroscience and Vascular Biology Laboratory, Department of Neurosurgery, University of Kentucky Medical Center, Lexington, Kentucky, USA; 2 Molecular and Cell Nutrition Laboratory, College of Agriculture, University of Kentucky, Lexington, Kentucky, USA

**Keywords:** blood–brain barrier, brain metastasis, polychlorinated biphenyls, tight junctions

## Abstract

**Background:**

Polychlorinated biphenyls (PCBs) comprise a ubiquitous class of toxic substances associated with carcinogenic and tumor-promoting effects as well as neurotoxic properties in the brain. However, the effects of PCBs on the development of tumor metastases are not fully understood.

**Objective:**

We evaluated the hypothesis that exposure to individual PCB congeners can facilitate the development of brain metastases in immunocompetent mice via the disruption of the integrity of the blood–brain barrier (BBB).

**Methods:**

C57/Bl6 mice were exposed to individual PCBs by oral gavage, and 48 hr later they were injected with luciferase-labeled K1735 M2 melanoma cells into the internal carotid artery. The development of metastatic nodules was monitored by bioluminescent imaging. In addition, we evaluated the functional permeability of the BBB by measuring permeability of sodium fluorescein across the brain microvessels. Expression and colocalization of tight junction (TJ) proteins were studied by Western blotting and immunofluorescence microscopy.

**Results:**

Oral administration of coplanar PCB126, mono-*ortho*-substituted PCB118, and non-coplanar PCB153 (each at 150 μmol/kg body weight) differentially altered expression of the TJ proteins claudin-5, occludin, and zonula occludens-1 in brain capillaries. These alterations were associated with increased permeability of the BBB. Most importantly, exposure to individual PCB congeners enhanced the rate of formation and progression of brain metastases of luciferase-tagged melanoma cells.

**Conclusions:**

Our results show for the first time that exposure to individual PCBs can facilitate the formation of bloodborne metastases via alterations of the integrity of the brain capillary endothelium.

Polychlorinated biphenyls (PCBs) are persistent environmental toxicants associated with numerous adverse health effects. The widespread commercial use of PCBs, which peaked in the 1970s, contributed to the pervasive bioaccumulation of these toxicants in the environment (Longnecker et al. 1997). Environmental exposure to PCBs is ongoing as a result of continued use and disposal of products containing these toxicants (Prince et al. 2006), widespread bioaccumulation of PCBs in the biosphere, and bioconcentration in the food chain (Norstrom et al. 2009). Meat and fish remain the primary source of PCB exposure for most of the adult human population and account for consistent PCB accumulation within human tissues (Schneider et al. 2007).

Several studies have evaluated associations of PCBs with human health effects and have demonstrated adverse reproductive (Leoni et al. 1989), developmental (Jorissen 2007), immunologic (Glynn et al. 2008), and neurologic (Prince et al. 2006) effects. The influence of PCBs on cancer risk is well established in animal studies (Glauert al. 2008; Lehmann et al. 2007); however, human epidemiology studies are less consistent. Several epidemiologic studies derived from retrospective mortality analysis have linked occupational PCB exposure with an increased risk of developing malignant melanoma and brain cancer (Hopf et al. 2009; Knerr and Schrenk 2006; Loomis et al. 1997; Prince et al. 2006; Robinson et al. 1999; Sinks et al. 1992). Interestingly, the risk of developing brain cancer has been shown to be independent of cumulative PCB exposure (Loomis et al. 1997; Sinks et al. 1992).

The ability of PCBs to accumulate in brain tissue (Caudle et al. 2006; Saghir et al. 2000; Sipka et al. 2008) is likely related to their neurotoxicity. However, less is known about the effects of PCBs on brain endothelium. Previous research from our laboratory has demonstrated that PCBs can modulate properties of brain endothelial cells *in vitro* and enhance adhesion and transendothelial migration of tumor cells (Choi et al. 2003; Eum et al. 2004, 2006). However, the interactions of PCBs with brain endothelium and the blood–brain barrier (BBB) *in vivo* are virtually unknown.

The BBB is anatomically situated at the level of the cerebral microvascular capillary endothelium and it regulates the blood–brain exchange. The tight junctions (TJs) limit passive paracellular movement of solutes, ions, and water across the BBB. They form the most apical element of the junctional complex and are composed of an intricate complex of transmembrane, accessory, and cytoplasmic proteins that connect the TJs to actin cytoskeleton and intracellular signaling systems (Abbott et al. 2006). The transmembrane proteins occludin and claudin-5 form the primary seal of the TJs. They bind to the intracellular proteins zonula occludens (ZO)-1, ZO-2, cingulin, and/or 7H6 that couple the TJs to the actin cytoskeleton of endothelial cells (Abbott et al. 2006). TJ proteins are also important in maintaining barrier polarity and are involved in cellular signaling. Disruption of the integrity of this system has been associated with several central nervous system pathologies (Weiss et al. 2009).

In the present study, we hypothesize that individual PCB congeners can influence the BBB integrity at the level of the TJs and contribute to the development of brain cancer metastasis. We demonstrate that exposure to coplanar PCB126, mono-*ortho*-substituted PCB118, and non-coplanar PCB153 can differentially alter expression of TJ proteins *in vivo*, induce permeability of brain microvessels, and stimulate formation of brain metastases of melanoma cells. Among the studied compounds, these changes appeared to be most advanced in animals exposed to PCB118.

## Materials and Methods

### Materials

We purchased PCB153 (2,2′,4,4′,5,5′-hexachlorobiphenyl), PCB126 (3,3′,4,4′,5-pentachlorobiphenyl), and PCB118 (2,3′,4,4′,5-pentachlorobiphenyl), all > 99% pure, from AccuStandard (New Haven, CT). Primary antibodies for occludin, ZO-1, and claudin-5 were obtained from Invitrogen (Camarillo, CA), and all secondary antibodies were purchased from Santa Cruz Biotechnology (Santa Cruz, CA). All other chemicals, unless otherwise stated, were purchased from Sigma (St. Louis, MO).

### Animals and PCB exposure

All animal procedures were approved by the University of Kentucky Animal Care and Use Committee in accordance with National Institutes of Health (NIH) guidelines (Institute for Laboratory Animal Resources 1996). The animals were treated humanely and with regard for alleviation of suffering. Studies were performed on male C57Bl/6 mice (Charles River Laboratories, Wilmington, MA), 14–16 weeks of age. Mice were gently restrained and administered via oral gavage with individual PCBs (75 or 150 μmol/kg body weight) dissolved in vitamin E–stripped safflower oil (Dyetts, Bethlehem, PA). PCBs are highly lipophilic compounds that are soluble in organic solvents. We chose safflower oil, a natural dietary component, as the PCB vehicle. Several biological effects of PCBs are mediated by oxidative stress; therefore, we used safflower oil that was stripped of vitamin E to avoid a possible influence of this antioxidant. Control animals were treated with safflower oil vehicle alone. Most of the experiments were performed 48 hr after PCB administration to mimic acute exposure characteristic of accidental poisoning with PCBs, such as the Yusho incident caused by ingestion of rice oil contaminated with PCBs (Shimizu et al. 2007). However, we analyzed brain metastasis formation for up to 9 days after PCB treatment.

### K1735 melanoma cell treatment and bioluminescent imaging

K1735 M2 murine melanoma cells transfected with a luciferase tag were a kind gift from I.J. Fidler (M.D. Anderson Cancer Center, Houston, TX). Cells were cultured in DMEM glutaMax medium (Invitrogen) supplemented with 10% fetal bovine serum. Mice were injected with 5 × 10^5^ cells in 100 μL phosphate-buffered saline (PBS) into the internal carotid artery as described previously by Chen et al. (2009). We monitored the development of metastatic nodules on days 4 and 7 after tumor cell injection using an IVIS Xenogen bioluminescence imager (Caliper LifeSciences, Hopkinton, MA). Animals were anesthetized by intraperitoneal (IP) injection with sodium pentobarbital (50 mg/kg) and treated with d-luciferin potassium salt (2 mg/100 μL, IP) to induce bioluminescence within proliferating K1735 cells. Identical instrument settings were used for all measurements to allow for adequate data comparisons. The experimental model is specific for the development of brain metastases. Nevertheless, approximately 2 weeks after tumor cell injection (i.e., past the time frame of the present study), we observed metastasis formation in peripheral organs, such as the lungs and liver, in selected animals (data not shown).

### Microvessel isolation

Brain microvessels were isolated as described previously (Seelbach et al. 2007). Under anesthesia, animals were perfused with saline; the brains were removed and immediately immersed in ice-cold isolation buffer A [103 mM sodium chloride (NaCl), 4.7 mM potassium chloride (KCl), 2.5 mM calcium chloride (CaCl_2_), 1.2 mM potassium phosphate (KH_2_PO_4_), 1.2 mM magnesium sulfate (MgSO_4_), and 15 mM HEPES; pH 7.4 with Complete Protease Inhibitor (Roche, Indianapolis, IN)]. We removed the choroid plexus, meninges, cerebellum, and brain stem, and homogenized the brains in 5 mL isolation buffer B [103 mM NaCl, 4.7 mM KCl, 2.5 mM CaCl_2_, 1.2 mM KH_2_PO_4_, 1.2 mM MgSO_4_, 15 mM HEPES, 25 mM NaHCO_3_, 10 mM glucose, 1 mM Na pyruvate, and dextran (molecular weight 64kD;10 g/L); pH 7.4] with Complete Protease Inhibitor. We then added 7 μL 26% dextran to the homogenates and centrifuged samples at 5,800 × *g* for 20 min. The supernatants were discarded, and the pellets were resuspended in 10 μL isolation buffer B and filtered through a 70-μm mesh filter (Becton Dickinson, Franklin, NJ). Filtered homogenates were repelleted by centrifugation and either smeared on slides for confocal analysis or resuspended in 150 μL of 6 M urea lysis buffer with Complete Protease Inhibitor for Western blot analyses. Diameters of isolated microvessels ranged from 4 to 10 μm.

### Western blotting and immunofluorescence microscopy

We performed Western blotting and immunofluorescence as described previously (Seelbach et al. 2007). We used anti-occludin antibody at a dilution of 1:1,000, anti-claudin-5 antibody at 1:500, anti-ZO-1 antibody at 1:500, and anti-actin antibody at 1:5,000. The proteins of interest were detected using the ECL Plus detection system (Amersham, Piscataway, NJ). Semiquantitation of protein was performed using NIH ImageJ software (Rasband 2009).

Freshly isolated intact microvessels were spread onto glass microscope slides and heat-fixed for 10 min at 95°C. All primary antibodies were diluted 1:500 in 1% bovine serum albumin in PBS. Slides were mounted with ProLong Gold Antifade reagent (Invitrogen) containing 4′,6-diamidino-2-phenylindole (DAPI) to visualize the nuclei. Images were acquired using an Olympus BX61WI laser scanning confocal microscope (Olympus, Melville, NY).

### BBB functional permeability assay

We assessed BBB permeability 48 hr after PCB administration using an adaptation of a previously described method (Chen et al. 2009). Briefly, animals were injected IP with 200 μL 10% sodium fluorescein that was allowed to circulate for 15 min, and blood was collected via cardiac puncture. Animals were then transcardially perfused with 0.9% saline and brains were harvested. Fluorescence was determined using excitation at 485 nm and emission at 530 nm. The permeability results are presented as a ratio of brain to plasma fluorescence intensity.

### Statistical analysis

We compared the treatment and vehicle control groups using one-way or two-way analysis of variance and performed the pairwise comparison using Student-Newman-Keuls post hoc test. Statistical probability of *p* < 0.05 was considered significant.

## Results

### TJ protein expression level in brain microvessels after oral administration of individual PCB congeners

In the first series of experiments, we investigated the influence of individual PCB congeners on the expression levels of key TJ proteins in brain capillaries. Because PCB exposure does not alter mRNA levels of genes encoding for TJ proteins (Eum et al. 2008), our studies focused on the effects of PCBs on protein expression of claudin-5, occludin, and ZO-1. These TJ proteins regulate the integrity and the barrier function of the BBB.

**Figure 1:**
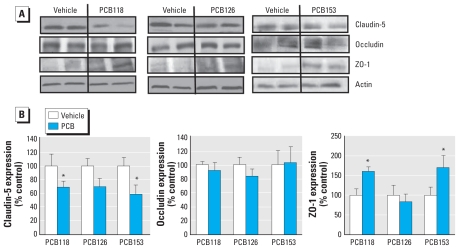
Treatment with individual PCB congeners differentially alters the expression of TJ proteins in cerebral microvessels of mice 48 hr after treatment with individual PCB congeners (150 μmol/kg) or vehicle. (*A*) Representative Western blots showing expression levels of claudin-5, occludin, and ZO-1. (*B*) Quantified results (mean ± SE ) of TJ protein expression obtained by densitometry analysis of Western blots and normalized to actin levels (*n* = 3–6 per group). *p < 0.05 compared with vehicle.

Forty-eight hours after administration of PCB118 or PCB153, we observed a significant decrease in the expression of claudin-5 compared with the vehicle control group. Figure 1A illustrates the representative images of Western blotting from these experiments; the quantified densitometry results are shown in Figure 1B. The changes in claudin-5 levels in PCB126-treated animals were not significant, despite demonstrating a strong trend toward a decrease. The expression level of ZO-1 was increased after treatment with PCB118 or PCB153, without any changes in PCB126-exposed animals. No alterations in the expression of occludin were detected after any PCB treatment.

### PCB-induced alterations of TJ immunofluorescence and immunolocalization

**Figure 2:**
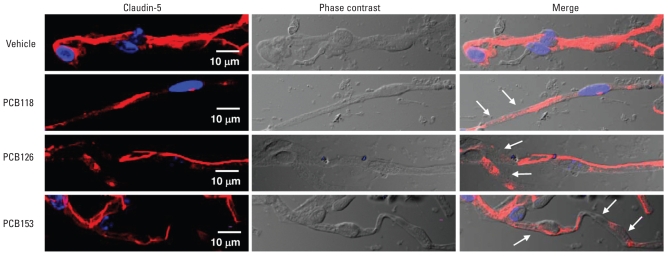
PCB exposure decreases claudin-5 immunoreactivity in cerebral microvessels in mice 48 hr after treatment with individual PCB congeners (150 μmol/kg) or vehicle. Images were acquired using a 60× oil-immersion lens and confocal microscopy. Claudin-5 immunoreactivity is stained in red; staining with DAPI was performed to visualize the nuclei (stained in blue); phase contrast micrographs illustrate the isolated microvessels; and merged micrographs show localized claudin-5 immunoreactivity within the microvessels. Arrows illustrate areas of disrupted claudin-5 immunoreactivity; *n* = 3–4 per group.

**Figure 3:**
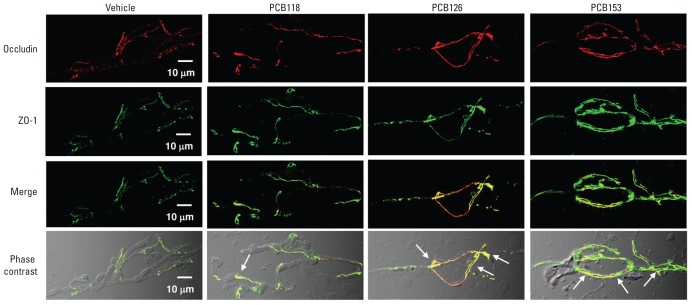
Individual PCB congeners differentially influence the staining intensity and association of occludin and ZO-1 in cerebral microvessels in mice 48 hr after treatment with PCB118, PCB126, or PCB153 (150 μmol/kg) compared with vehicle controls. Occludin (red) and ZO-1 (green) immunoreactivity was evaluated using confocal microscopy, and images were acquired using a 60× oil-immersion lens. Merged micrographs were obtained by superimposing images of the corresponding optical sections stained for occludin and ZO-1, and phase contrast micrographs show localized TJ immunoreactivity within the brain microvessels. Regions of occludin and ZO-1 colocalization are depicted in yellow (see arrows); *n* = 6 per group.

The integrity of the BBB is influenced not only by the expression level of TJ proteins but also by their cellular locations. Therefore, we examined the effects of PCB treatment on the localization profiles of claudin-5, occludin, and ZO-1 in freshly isolated microvessels (Figures 2 and 3). A semiqualitative analysis by immunofluorescence microscopy indicated that treatment with PCBs decreased the intensity and disrupted the continuity of claudin-5 immunoreactivity, similar to results of Western blotting. These alterations were visible in all PCB treatment groups we examined (Figure 2, arrows).

Compared with occludin, the immunostaining results for ZO-1 suggest a qualitative increase in immunoreactivity after treatment with individual PCB congeners (Figure 3). Because occludin interacts with ZO-1 at its C-terminus, we next determined the effects of individual PCB congeners on colocalization staining patterns for these proteins. Confocal microscopy suggests that PCB treatment can enhance the association between ZO-1 and occludin, as shown in yellow in the merged and phase-contrast micrographs in Figure 3 (arrows). The enhanced interaction between ZO-1 and occludin appeared to be most pronounced in cerebral microvessels of PCB126-treated animals, followed by the PCB153 treatment group.

### Oral administration of PCBs disrupts BBB integrity

**Figure 4:**
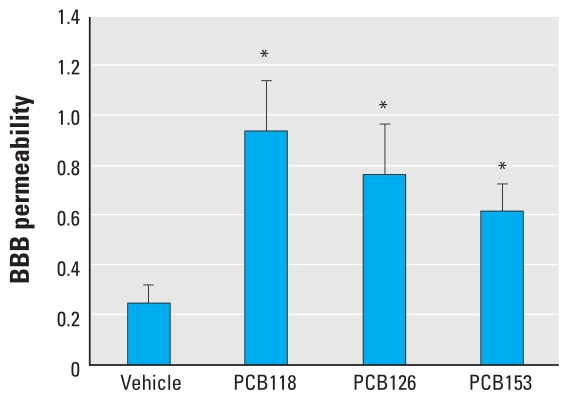
Exposure to PCBs increases BBB permeability. Mice were treated with individual PCB congeners (150 μmol/kg) or vehicle for 48 hr. Then, 200 μL of 10% sodium fluorescein was injected IP and allowed to circulate for 15 min. The ratio of brain to plasma sodium fluorescence level serves as a marker of brain permeability; results are mean ± SE (*n* = 5–6 per group). *p < 0.05 compared with vehicle.

Alterations of TJ molecular properties have been associated with alterations of BBB integrity (Abbott et al. 2006). Therefore, we evaluated the effects of individual PCB congeners on permeability of brain microvessels using a low-molecular tracer, sodium fluorescein. Forty-eight hours after PCB treatment (150 μmol/kg), we observed a statistically significant increase in sodium fluorescein permeability across the capillary endothelium in all treatment groups (Figure 4). The most pronounced permeability changes were observed in the PCB118 treatment group.

### Administration of PCBs enhances the formation of brain metastases

**Figure 5:**
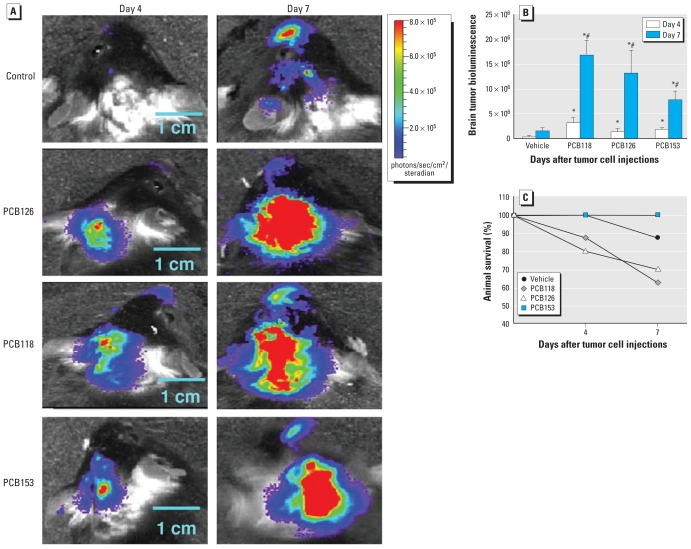
The rate and progression of brain metastasis are enhanced in mice exposed to individual PCB congeners. Mice were treated with PCBs (150 μmol/kg) or vehicle for 48 hr and then injected with K1735-luciferase–tagged melanoma cells (5 × 10^5^ cells in 100 μL) to the internal carotid artery. Growth of brain metastases was measured based on the intensity of the bioluminescent signal. (*A*) Representative images of brains of the animals at days 4 and 7 postinjection of K1735 cells normalized to the same color scale. (*B*) Quantified results of brain tumor bioluminescence indicating the progression of brain metastasis growth. (*C*) Animal survival rates for up to 7 days postinjection of K1735 cells. For *B* and *C*, results are mean ± SE; *n* = 3–6 per group. *p < 0.05 compared with the vehicle-treated group at the same day after treatment. ^#^*p* < 0.05 compared with the corresponding treatment at day 4.

We next investigated the influence of individual PCB congeners on the growth rate and progression of brain metastases. Mice were administered individual PCB congeners by oral gavage, followed by injection with melanoma cells into the internal carotid artery 48 hr later. We measured metastatic tumor growth 4 and 7 days after tumor cell injection, that is, at the time points when initial metastatic nodules have been established (day 4) and fully developed (day 7). As illustrated in Figure 5A, the growth of metastatic tumors in the control group was minimal at day 4 after tumor cell injection. However, at day 7 the control mice developed substantial bioluminescence corresponding to metastatic tumors. Compared with controls, PCB-treated mice displayed a marked increase in brain metastasis (Figure 5A, B). Brain tumor bioluminescence images demonstrated a robust tumor growth response for all PCB-treated animals. These changes were the most evident 7 days after tumor cell injection. Animals exposed to PCB118 and PCB126 displayed relatively greater susceptibility to tumor growth compared with PCB153 (Figure 5A, B). Likewise, survival rate after tumor cell administration was lowest for PCB126- and PCB118-treated animals (Figure 5C).

**Figure 6:**
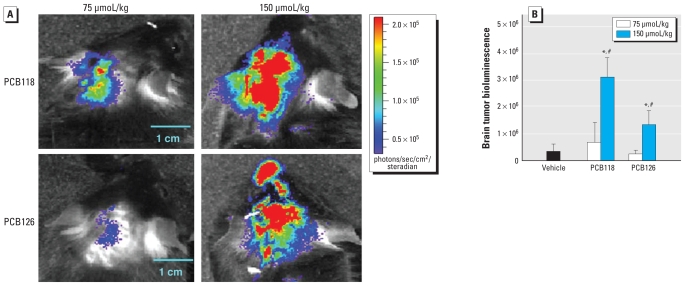
PCBs enhance rate and progression of brain metastasis in a dose-dependent manner. Mice were treated with PCB118 or PCB126 (75 or 150 μmol/kg) or vehicle by oral gavage. After 48 hr, mice were injected with K1735-luciferase–tagged melanoma cells, and the progression of brain metastases was assessed at day 4 after tumor cell injection. (*A*) Representative images of brains normalized to the same color scale. (*B*) Quantified results of brain tumor bioluminescence; results are mean ± SE; *n* = 4–6 per group. *p < 0.05 compared with the vehicle-treated group. ^#^*p* < 0.05 compared with the 75-μmol/kg treatment group.

We also evaluated the dose-dependent effects of PCBs on the development of brain metastases. Because of the stimulatory effects of PCB118 and PCB126 on the development of brain metastases, we included these PCBs at 75 and 150 μmol/kg in our experiments. The measurements of metastatic growth were performed at day 4 after injection of tumor cells. Compared with controls, PCB118 and PCB126 (75 μmol/kg) did not influence the magnitude of brain metastasis initiation or progression (Figure 6). Consistent with the results in Figure 5, only a higher dose of PCBs (150 μmol/kg) markedly stimulated brain metastasis formation as determined by tumor bioluminescence values.

## Discussion

A link between PCB exposure and pathology has been strongly established in the literature (Hennig et al. 2005; Safe 1989). Both early childhood development and adult neurologic functions may be impaired by PCB exposure (Faroon et al. 2001; Grandjean et al. 2001; Petersen et al. 2008). For example, a cohort analysis study performed on 7-year-old children showed that, in conjunction with mercury exposure, high umbilical cord PCB levels augmented neurobehavioral deficits later in life (Grandjean et al. 2001). Further, epidemiologic studies support an association between PCB exposure and central nervous system disease, including Parkinson disease, amyotrophic lateral sclerosis, non–Alzheimer-related dementia, and brain cancer in adults (Caudle et al. 2006; Hopf et al. 2009; Steenland et al. 2006). The BBB breakdown is a commonality in all of these central nervous system disease states (Weiss et al. 2009); however, the pathophysiologic influence of PCBs on the BBB function is not fully understood.

Research from our laboratory indicated that selected PCB congeners can stimulate proinflammatory properties (Eum et al. 2004, 2006). These studies linked PCB exposure with prometastatic changes of cultured endothelial cells. Indeed, we demonstrated that exposure of endothelial cells to *ortho*-substituted PCBs can stimulate production of inflammatory mediators leading to adhesion and transmigration of THP-1 or breast cancer cells (MDA-MB-231) (Choi et al. 2003; Eum et al. 2004). It appears that activation of JAK3, EGFR, Src kinase, and MAP kinase signaling cascades may underlie these effects (Eum et al. 2004, 2006). However, transendothelial migration of tumor cells may be further potentiated by disruption of endothelial cell junctions and hyperpermeability across the endothelial barrier. Therefore, in the present study we explored the influence of both coplanar and noncoplanar PCBs on molecular and functional properties of the BBB. We focused on three typical PCB congeners that maintain different structural properties, namely, PCB153, PCB126, and PCB118. Noncoplanar PCB153 accounts for the majority of PCBs found in environmental and in biological samples (Faroon et al. 2001). PCB126 belongs to the coplanar dioxin-like group of PCBs that act through an aryl hydrocarbon receptor (AhR) mechanism. In contrast, mono-*ortho*-substituted PCB118 is a weak AhR agonist (Hestermann et al. 2000).

In the present study, the molecular analysis of TJ proteins indicated that PCBs can differentially modulate TJ expression and/or localization. Exposure to PCB118 and PCB153 modulated expressional levels of claudin-5 and ZO-1, whereas PCB126 appeared to primarily influence the coassociation of occludin and ZO-1. These findings suggest that PCB congeners may be acting through different mechanisms to elicit the same functional effect (i.e., disrupted barrier integrity and increased permeability across the endothelial clefts). The precise mechanisms responsible for these events have yet to be elucidated; however, they are likely to involve redox-responsive reactions. Indeed, PCBs are potent generators of reactive oxygen species (Choi et al. 2003; Hassoun et al. 2002; Hennig et al. 2002), and the alterations of redox signaling contribute to the disruption of the BBB function (Haorah et al. 2007; Persidsky et al. 2006). The brain is highly sensitive to oxidative stress because of its high oxygen consumption, high iron and lipid content, and low activity for antioxidant defenses. Studies performed in our laboratory (András et al. 2005; Zhong et al. 2008) and others (Haorah et al. 2007; Persidsky et al. 2006) demonstrate that oxidative stress can alter the integrity of the BBB at the level of the TJs acting through Ras and Rho redox-responsive elements. The Ras GTPase pathway plays an important role in the regulation of claudin-5, ZO-1, and ZO-2 (Zhong et al. 2008), and the Rho pathway is critical for TJ assembly (Persidsky et al. 2006). Other redox-regulated candidate pathways that may be involved in PCB-induced alterations of TJ protein expression include MAP kinase (Krizbai et al. 2005) and PI3K (Schreibelt et al. 2007). Finally, these mechanisms may involve proteolysis of TJ proteins by matrix metalloproteinases (MMPs) and ubiquitination-proteasome systems (Eum et al. 2008).

One of the major goals of the present study was to develop a model to evaluate the effects of PCBs on the development of tumor metastases. We focused on brain metastases of melanoma cells, because metastatic brain tumors originating from malignant melanoma are seen frequently in clinical medicine (Fidler et al. 1999; Gonzalez-Martinez et al. 2002). Over the last 30 years, the mortality rate of malignant melanomas has increased by 50% (Geller et al. 2002). Using a modification of previously published methods (Chen et al. 2009; Fidler et al. 1999; Zhang et al. 2008), we introduced the highly metastatic melanoma cells into the internal carotid artery of fully immunocompetent mice. Because the internal carotid artery supplies the brain parenchyma with blood, tumor cell injection into brain vasculature mimics the metastatic process in humans in which circulating tumor cells adhere and invade capillary endothelium (Zhang et al. 2008). Our newly developed model resulted in the consistent development of metastatic nodules within brain parenchyma, an effect that was highly potentiated by pre-exposure to individual PCB congeners, especially PCB118. Several factors participate in the propensity of malignant melanoma to selectively target brain tissue (Palmieri et al. 2007; Rolland et al. 2009); however, tumor cell extravasation into the brain is dependent upon its interactions at the cerebral capillary endothelium. Thus, the leaky BBB due to PCB-induced alterations of TJ expression in brain microvessels may facilitate transcapillary transfer of tumor cells and contribute to the development of brain metastasis.

## Conclusion

The present study indicates that exposure to PCBs leads to disruption of the BBB integrity via modulation of TJ protein expression. Most important, we demonstrate for the first time that oral exposure to specific PCB congeners can enhance the rate of brain metastasis growth. These results suggest that alteration of BBB integrity is the underlying mechanism of PCB-induced brain metastases and may also be involved in neurotoxic and neurodevelopmental effects of these environmental toxicants.

## References

[b1-ehp-118-479] Abbott NJ, Ronnback L, Hansson E (2006). Astrocyte-endothelial interactions at the blood-brain barrier. Nat Rev Neurosci.

[b2-ehp-118-479] András IE, Pu H, Tian J, Deli MA, Nath A, Hennig B (2005). Signaling mechanisms of HIV-1 Tat-induced alterations of claudin-5 expression in brain endothelial cells. J Cereb Blood Flow Metab.

[b3-ehp-118-479] Caudle WM, Richardson JR, Delea KC, Guillot TS, Wang M, Pennell KD (2006). Polychlorinated biphenyl-induced reduction of dopamine transporter expression as a precursor to Parkinson’s disease-associated dopamine toxicity. Toxicol Sci.

[b4-ehp-118-479] Chen L, Swartz KR, Toborek M (2009). Vessel microport technique for applications in cerebrovascular research. J Neurosci Res.

[b5-ehp-118-479] Choi W, Eum SY, Lee YW, Hennig B, Robertson LW, Toborek M (2003). PCB 104-induced proinflammatory reactions in human vascular endothelial cells: relationship to cancer metastasis and atherogenesis. Toxicol Sci.

[b6-ehp-118-479] Eum SY, András IE, Couraud PO, Hennig B, Toborek M (2008). PCBs and tight junction expression. Environ Toxicol Pharmacol.

[b7-ehp-118-479] Eum SY, Lee YW, Hennig B, Toborek M (2004). VEGF regulates PCB 104-mediated stimulation of permeability and transmigration of breast cancer cells in human microvascular endothelial cells. Exp Cell Res.

[b8-ehp-118-479] Eum SY, Lee YW, Hennig B, Toborek M (2006). Interplay between epidermal growth factor receptor and Janus kinase 3 regulates polychlorinated biphenyl-induced matrix metalloproteinase-3 expression and transendothelial migration of tumor cells. Mol Cancer Res.

[b9-ehp-118-479] Faroon O, Jones D, de Rosa C (2001). Effects of polychlorinated biphenyls on the nervous system. Toxicol Ind Health.

[b10-ehp-118-479] Fidler IJ, Schackert G, Zhang RD, Radinsky R, Fujimaki T (1999). The biology of melanoma brain metastasis. Cancer Metastasis Rev.

[b11-ehp-118-479] Geller AC, Miller DR, Annas GD, Demierre MF, Gilchrest BA, Koh HK (2002). Melanoma incidence and mortality among US whites, 1969–1999. JAMA.

[b12-ehp-118-479] Glauert HP, Tharappel JC, Banerjee S, Chan NL, Kania-Korwel I, Lehmler HJ (2008). Inhibition of the promotion of hepatocarcinogenesis by 2,2′,4,4′,5,5′-hexachlorobiphenyl (PCB-153) by the deletion of the p50 subunit of NF-kappa B in mice. Toxicol Appl Pharmacol.

[b13-ehp-118-479] Glynn A, Thuvander A, Aune M, Johannisson A, Darnerud PO, Ronquist G (2008). Immune cell counts and risks of respiratory infections among infants exposed pre- and postnatally to organochlorine compounds: a prospective study. Environ Health.

[b14-ehp-118-479] Gonzalez-Martinez J, Hernandez L, Zamorano L, Sloan A, Levin K, Lo S (2002). Gamma knife radiosurgery for intracranial metastatic melanoma: a 6-year experience. J Neurosurg.

[b15-ehp-118-479] Grandjean P, Weihe P, Burse VW, Needham LL, Storr-Hansen E, Heinzow B (2001). Neurobehavioral deficits associated with PCB in 7-year-old children prenatally exposed to seafood neurotoxicants. Neurotoxicol Teratol.

[b16-ehp-118-479] Haorah J, Ramirez SH, Schall K, Smith D, Pandya R, Persidsky Y (2007). Oxidative stress activates protein tyrosine kinase and matrix metalloproteinases leading to blood-brain barrier dysfunction. J Neurochem.

[b17-ehp-118-479] Hassoun EA, Wang H, Abushaban A, Stohs SJ (2002). Induction of oxidative stress in the tissues of rats after chronic exposure to TCDD, 2,3,4,7,8-pentachlorodibenzofuran, and 3,3′,4,4′,5-pentachlorobiphenyl. J Toxicol Environ Health A.

[b18-ehp-118-479] Hennig B, Meerarani P, Slim R, Toborek M, Daugherty A, Silverstone AE (2002). Proinflammatory properties of coplanar PCBs: in vitro and in vivo evidence. Toxicol Appl Pharmacol.

[b19-ehp-118-479] Hennig B, Reiterer G, Majkova Z, Oesterling E, Meerarani P, Toborek M (2005). Modification of environmental toxicity by nutrients: implications in atherosclerosis. Cardiovasc Toxicol.

[b20-ehp-118-479] Hestermann EV, Stegeman JJ, Hahn ME (2000). Relative contributions of affinity and intrinsic efficacy to aryl hydrocarbon receptor ligand potency. Toxicol Appl Pharmacol.

[b21-ehp-118-479] Hopf NB, Waters MA, Ruder AM (2009). Cumulative exposure estimates for polychlorinated biphenyls using a job-exposure matrix. Chemosphere.

[b22-ehp-118-479] Institute for Laboratory Animal Resources (1996). NIH Guide for the Care and Use of Laboratory Animals.

[b23-ehp-118-479] Jorissen J (2007). Literature review. Outcomes associated with postnatal exposure to polychlorinated biphenyls (PCBs) via breast milk. Adv Neonatal Care.

[b24-ehp-118-479] Knerr S, Schrenk D (2006). Carcinogenicity of “non-dioxinlike” polychlorinated biphenyls. Crit Rev Toxicol.

[b25-ehp-118-479] Krizbai IA, Bauer H, Bresgen N, Eckl PM, Farkas A, Szatmári E (2005). Effect of oxidative stress on the junctional proteins of cultured cerebral endothelial cells. Cell Mol Neurobiol.

[b26-ehp-118-479] Lehmann L, Esch HL, Kirby PA, Robertson LW, Ludewig G (2007). 4-Monochlorobiphenyl (PCB3) induces mutations in the livers of transgenic Fisher 344 rats. Carcinogenesis.

[b27-ehp-118-479] Leoni V, Fabiani L, Marinelli G, Puccetti G, Tarsitani GF, De Carolis A (1989). PCB and other organochlorine compounds in blood of women with or without miscarriage: a hypothesis of correlation. Ecotoxicol Environ Saf.

[b28-ehp-118-479] Longnecker MP, Rogan WJ, Lucier G (1997). The human health effects of DDT (dichlorodiphenyltrichloroethane) and PCBS (polychlorinated biphenyls) and an overview of organochlorines in public health. Annu Rev Public Health.

[b29-ehp-118-479] Loomis D, Browning SR, Schenck AP, Gregory E, Savitz DA (1997). Cancer mortality among electric utility workers exposed to polychlorinated biphenyls. Occup Environ Med.

[b30-ehp-118-479] Norstrom K, Czub G, McLachlan MS, Hu D, Thorne PS, Hornbuckle KC (2009). External exposure and bioaccumulation of PCBs in humans living in a contaminated urban environment. Environ Int.

[b31-ehp-118-479] Palmieri D, Chambers AF, Felding-Habermann B, Huang S, Steeg PS (2007). The biology of metastasis to a sanctuary site. Clin Cancer Res.

[b32-ehp-118-479] Persidsky Y, Heilman D, Haorah J, Zelivyanskaya M, Persidsky R, Weber GA (2006). Rho-mediated regulation of tight junctions during monocyte migration across the blood–brain barrier in HIV-1 encephalitis (HIVE). Blood.

[b33-ehp-118-479] Petersen MS, Halling J, Bech S, Wermuth L, Weihe P, Nielsen F (2008). Impact of dietary exposure to food contaminants on the risk of Parkinson’s disease. Neurotoxicology.

[b34-ehp-118-479] Prince MM, Ruder AM, Hein MJ, Waters MA, Whelan EA, Nilsen N (2006). Mortality and exposure response among 14,458 electrical capacitor manufacturing workers exposed to polychlorinated biphenyls (PCBs). Environ Health Perspect.

[b35-ehp-118-479] Rasband WS (2009). ImageJ.

[b36-ehp-118-479] Robinson CF, Petersen M, Palu S (1999). Mortality patterns among electrical workers employed in the U.S. construction industry, 1982–1987. Am J Ind Med.

[b37-ehp-118-479] Rolland Y, Demeule M, Fenart L, Béliveau R (2009). Inhibition of melanoma brain metastasis by targeting melanotransferrin at the cell surface. Pigment Cell Melanoma Res.

[b38-ehp-118-479] Safe S (1989). Polychlorinated biphenyls (PCBs): mutagenicity and carcinogenicity. Mutat Res.

[b39-ehp-118-479] Saghir SA, Hansen LG, Holmes KR, Kodavanti PR (2000). Differential and non-uniform tissue and brain distribution of two distinct 14C-hexachlorobiphenyls in weanling rats. Toxicol Sci.

[b40-ehp-118-479] Schneider AR, Porter ET, Baker JE (2007). Polychlorinated biphenyl release from resuspended Hudson River sediment. Environ Sci Technol.

[b41-ehp-118-479] Schreibelt G, Kooij G, Reijerkerk A, van Doorn R, Gringhuis SI, van der Pol S (2007). Reactive oxygen species alter brain endothelial tight junction dynamics via RhoA, PI3 kinase, and PKB signaling. FASEB J.

[b42-ehp-118-479] Seelbach MJ, Brooks TA, Egleton RD, Davis TP (2007). Peripheral inflammatory hyperalgesia modulates morphine delivery to the brain: a role for P-glycoprotein. J Neurochem.

[b43-ehp-118-479] Shimizu K, Ogawa F, Thiele JJ, Bae S, Sato S (2007). Lipid peroxidation is enhanced in Yusho victims 35 years after accidental poisoning with polychlorinated biphenyls in Nagasaki, Japan. J Appl Toxicol.

[b44-ehp-118-479] Sinks T, Steele G, Smith AB, Watkins K, Shults RA (1992). Mortality among workers exposed to polychlorinated biphenyls. Am J Epidemiol.

[b45-ehp-118-479] Sipka S, Eum SY, Son KW, Xu S, Gavalas VG, Hennig B (2008). Oral administration of PCBs induces proinflammatory and prometastatic responses. Environ Toxicol Pharmacol.

[b46-ehp-118-479] Steenland K, Hein MJ, Cassinelli RT, Prince MM, Nilsen NB, Whelan EA (2006). Polychlorinated biphenyls and neurodegenerative disease mortality in an occupational cohort. Epidemiology.

[b47-ehp-118-479] Weiss N, Miller F, Cazaubon S, Couraud PO (2009). The blood–brain barrier in brain homeostasis and neurological diseases. Biochim Biophys Acta.

[b48-ehp-118-479] Zhang Z, Hatori T, Nonaka H (2008). An experimental model of brain metastasis of lung carcinoma. Neuropathology.

[b49-ehp-118-479] Zhong Y, Smart EJ, Weksler B, Couraud PO, Hennig B, Toborek M (2008). Caveolin-1 regulates human immunodeficiency virus-1 Tat-induced alterations of tight junction protein expression via modulation of the Ras signaling. J Neurosci.

